# Efficacy of Cord Blood Cell Therapy for Hutchinson–Gilford Progeria Syndrome—A Case Report

**DOI:** 10.3390/ijms222212316

**Published:** 2021-11-15

**Authors:** Mi Ri Suh, Ikhyun Lim, Jongwook Kim, Pil-Sung Yang, Jin Seung Choung, Hye Ryeong Sim, Sung Chan Ha, MinYoung Kim

**Affiliations:** 1 Department of Rehabilitation Medicine, CHA Bundang Medical Center, CHA University School of Medicine, Seongnam 13496, Korea; suhmiri@chamc.co.kr (M.R.S.); a196044@chamc.co.kr (I.L.); a186049@chamc.co.kr (J.K.); 2 Rehabilitation and Regeneration Research Center, CHA University School of Medicine, Seongnam 13488, Korea; choungjs@gmail.com (J.S.C.); ryeong1436@naver.com (H.R.S.); tjdcks6101@naver.com (S.C.H.); 3 Division of Cardiology, Department of Internal Medicine, CHA Bundang Medical Center, CHA University School of Medicine, Seongnam 13496, Korea; psyang01@cha.ac.kr

**Keywords:** cord blood cell therapy, Hutchinson–Gilford progeria syndrome, inflammation, atherosclerosis

## Abstract

Hutchinson–Gilford progeria syndrome (HGPS) is an extremely rare premature aging disorder characterized by short stature and atherosclerosis-induced death within teenage years. A 13-year-old male diagnosed with HGPS was administered three intravenous infusions of allogeneic cord blood (CB) cells from unrelated donors at four-month intervals to evaluate the safety and its therapeutic efficacy. Adverse events were monitored in addition to height, weight, laboratory blood tests, joint range of motion (ROM), and carotid Doppler. Cytokine and receptor assays were also performed. The patient exhibited an increase in growth rate for both height and weight. One year after therapy initiation, evident amelioration in pulse wave velocity, bilateral maximal intima-media thickness, and dyslipidemic status were observed, which were in abrupt aggravation prior to treatment. Further, an increase in flexibility occurred in some joints of the upper extremities. No serious adverse events were observed throughout the study period and one year beyond. A molecular assay revealed downregulation of proinflammatory and atherosclerosis, representing cytokine expressions following the administration of CB cells. This is the first reported case of an allogeneic CB trial in a patient with HGPS showing therapeutic effects of CB with improvements in anthropometric measures, joint ROM with amelioration of atherosclerosis, and dyslipidemia induced by anti-inflammatory and anti-atherosclerotic responses.

## 1. Introduction

Hutchinson–Gilford progeria syndrome (HGPS) is an extremely rare, premature aging disorder caused by mutations in the *LMNA* gene or abnormal posttranslational processing, which leads to the abnormal formation of lamin A protein. Most of the cases occur by a single nucleotide mutation (c.1824C > T, p.G608G) that activates a cryptic splice within exon 11 of *LMNA.* Following generation of an alternative lamin A transcript with an internal deletion of 150 nucleotides in mRNA, truncated protein lacking the 50-residue region that contains ZMPSTE24 cleavage site is synthesized [1]. Due to the absence of the ZMPSTE24 enzyme that is essential in the maturation of prelamin A, progerin, the farnesylated-prelamin A, accumulates and disturbs the organization of the nuclear lamina, which is responsible for the clinical manifestations [2]. These include extremely short stature, low body weight, early loss of hair, lipodystrophy, scleroderma-like skin, typical facial features, multiple joint contractures, and atherosclerosis [3,4,5]. Accumulation of progerin in endothelial cells leads to endothelial dysfunction [6], which then causes pathologic findings in the vasculature that resembles that of the elderly. Although cardiovascular problems are variable, both in the age of onset and nature, the symptoms arise around the age of 6–8, and strokes have been reported at a median of 9 years (4–19 years). Without treatment, death usually occurs within teenage years, at an average of 14.6 years, due to stroke and cardiovascular events, which are consequences of accelerated atherosclerosis [4,7].

Therapeutic measures for HGPS have been studied, which include medications purposed to inhibit synthesis, increase the clearance and reduce the accumulation of progerin [8,9]. Lonafarnib, a farnesyltransferase inhibitor, is currently the only approved drug for the treatment of HGPS. Its use with sirolimus has exhibited the most promising outcomes, with improved carotid artery pulse wave velocity (PWV) and a decline in mortality [10,11,12,13]. However, children who cannot tolerate the side effects of lonafarnib such as nausea, vomiting, diarrhea, infection, decreased appetite, and fatigue [12], or those with limited access to the medication due to its expense (average USD 1,032,480/year) [14], have no alternative options.

In progeria, disrupted nuclear membrane structural integrity affects cells consisting of skin and vasculature connective tissues. The affected blood vessel wall thickens while the lumen narrows, leading to increased arterial PWV. Both elderly atherosclerosis and HGPS share common outcomes of calcification, arterial wall inflammation, and plaque in the early to late-stage spectrum. HGPS lesions are similar to those of adult cardiovascular disease, exhibiting atherosclerotic lesion development and in situ inflammatory processes [15,16]. Bidault et al. also suggested the proatherogenic role of progerin in HGPS-related early atherosclerosis, where overexpression of which increases inflammation along with oxidative stress by increased expression of the proinflammatory cytokines interleukin-6 (IL-6) and interleukin-1β (IL-1β), and adhesion molecules intercellular adhesion molecule-1 (ICAM-1) and vascular cell adhesion molecule-1 [6]. Arteries are mainly afflicted, and arteriopathies in the cardiovascular and cerebrovascular systems result in fatal outcomes in teenagers. Stem cells have shown protective effects against atherosclerotic diseases by reducing serum lipid levels and inflammation and increasing plaque stability [17]. In addition, considering the angiogenic effect reported in previous studies [18,19] and immunologic responses [20], cell therapy could offer a new therapeutic avenue for this rare disease.

Cord blood (CB) as a therapeutic option is advantageous for its safety and immune tolerance [21] and has worldwide “bank” availability [22,23]. Allogeneic CB has shown possible therapeutic efficacy in clinical settings for children with cerebral palsy [24]. A clinical trial for adult strokes also yielded favorable outcomes with anti-inflammatory effects [24,25]. Therefore, we hypothesized that allogeneic CB, with a similar mechanism of action, might be beneficial to patients with HGPS. To the best of our knowledge, this is the first trial of allogeneic CB therapy for HGPS that analyzed clinical outcomes and therapeutic mechanisms.

## 2. Results

### 2.1. Adverse Events and Deviations from the Protocol

The patient did not show any serious adverse events during the study period of 12 months after the first infusion and until additional 12 months beyond the study period. He reported six adverse events during the study period: two fever events and one event each of common cold, chilling sensation, headache, and left ankle pain (Figure S1). All symptoms, except the left ankle pain at the malleolus, recovered spontaneously. All events were not considered related to the CB therapy as they occurred more than one month apart from each injection. Although the left ankle pain persisted, the X-ray and ultrasound evaluation did not reveal pathological problems; thus, it was considered to originate from the ankle contracture of the patient.

While oral sirolimus administration was planned, the patient arbitrarily took it for only three days (D−1 to D+1) upon the first infusion, two days (D−1 to D0) upon the second infusion, and none upon the third infusion. This was discovered after trial completion. While gentle stretching and regular exercises were recommended, his activity-related lifestyle was the same as before therapy.

### 2.2. Anthropometric, Musculoskeletal, and Cognitive Changes after CB Administration

**Figure 1:**
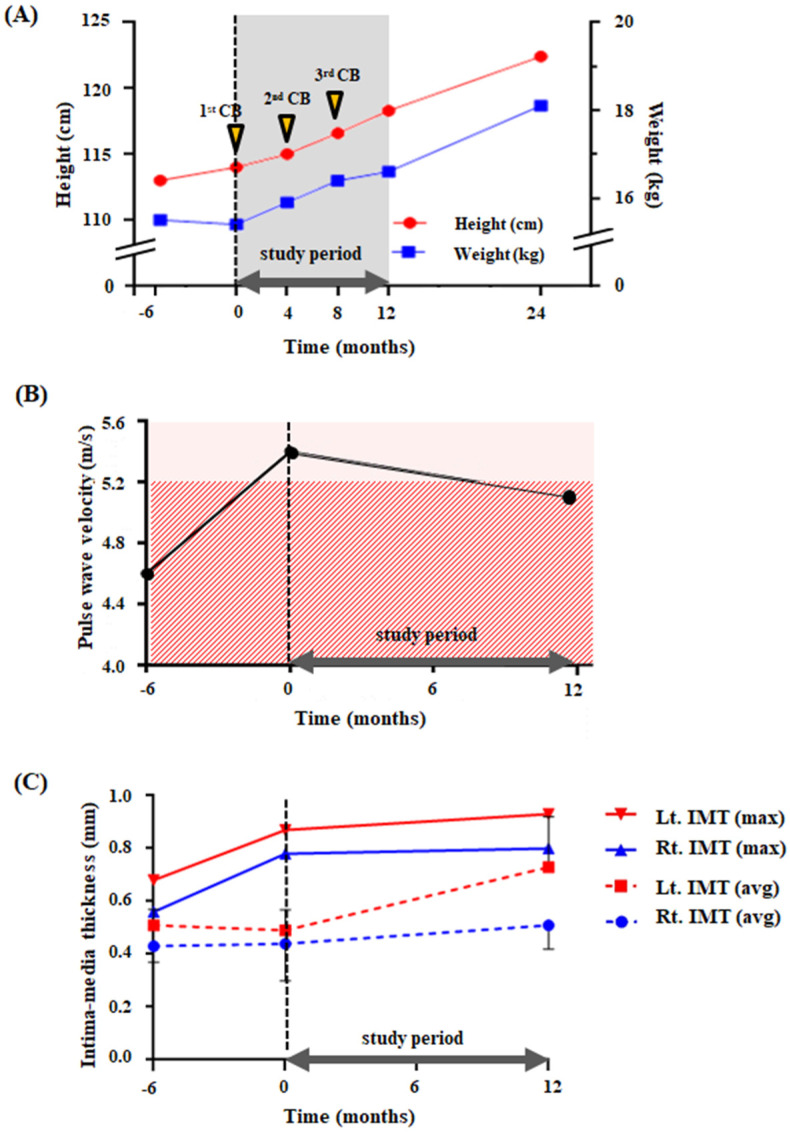
Changes before and after the cord blood cell therapy. (**A**) the height and the weight, (**B**) the changes of PWV, and (**C**) the changes of IMT through the study period. The dotted vertical line (0 months) shows the initiation of the trial. (**A**) The red dots show the height, and the blue dots show the weight of the patient. The yellow arrow represents each cord blood infusion, gray shaded area represents the 12 months of trial and follow-up period. (**B**) Pink shaded area represents the published normal range of PWV (3.9–6.3 m/s) at age 13–14. The red slashed area represents the normal range of PWV (3.5–5.2 m/s) for those whose height is 120 cm [26]. (**C**) The red and blue lines represent maximal IMT of left and right sides, respectively. The red and blue dotted lines represent average IMT of left and right sides, respectively. Error bars indicate standard deviation of each average IMT value. Thick bold line with arrows at both ends represents the 12 months of study period starting from the first infusion. Abbreviations: CB, cord blood; PWV, pulse wave velocity; IMT, intima-media thickness.

The patient constantly showed growth increase during the trial. His height was 113.0 cm six months before the trial, 114.0 cm on the date of first CB infusion, and he grew up to 118.3 cm one year after the first infusion (Figure 1A). His weight was initially 15.5 kg six months before therapy, slightly decreased to 15.4 kg immediately before therapy, and increased to 16.6 kg one year after the first injection. Height and weight increased to 122.4 cm and 18.1 kg, respectively, two years after the first infusion, which was 12 months beyond the original study period. His body mass index seemed to remain consistent throughout the study period, ranging between 11.8 and 12.2 kg/m^2^; 11.8 kg/m^2^ at the time of study initiation, just before the first CB infusion, and 11.9 kg/m^2^ one year after that time. Six months before the first infusion, it was 12.1 kg/m^2^, and two years after the first infusion, it was 12.2 kg/m^2^.

The ROM in many joints, including the shoulder, elbow, wrist, fingers, hip, knee, and ankle, were mildly limited at the first visit. After therapy, small increments of ROM were detected in the shoulders and elbows, while small decrements in ROM were detected in the knees and ankles (Table S3). Bone mineral density showed slightly worsened z-scores from −3.0 (L2) to −3.8 (L2) during the study period of 12 months. Cognitive function exhibited slight improvements with an increase in intelligence quotient (IQ) from 83 to 91.

### 2.3. Amelioration of Atherosclerotic Condition after CB Administration

The carotid Doppler study revealed several important findings. The baseline PWV six months before the therapy was 4.6 m/s, and it increased to 5.4 m/s immediately prior to the therapy. The value of 5.4 m/s was higher than the reference range (3.5–5.2 m/s) for the lowest height given (120 cm), which was the closest value to his actual height (115 cm) [26,27]. One year after the first infusion, the PWV decreased to 5.1 m/s, which was within the reference range (Figure 1B).

Average intima-media thickness (IMT) increased with age, mostly above the reference range for his age (0.4–0.5 mm) [28]. However, maximal IMT, which demonstrated steep increments (+0.037 mm/month and +0.032 mm/month for right and left side, respectively) before CB administration, showed a markedly decreased rate of increment (−0.002 mm/month and −0.005 mm/month for right and left side, respectively) on both sides after therapy (Figure 1C). The ankle-brachial index (ABI) remained within the reference range (0.77–1.15 m/s) [29] throughout the study period (Figure S2).

Laboratory blood test results showed rising triglyceride levels during the one month period before the first infusion, from 71 to 226 mg/dL, and continuous decrements later to the borderline values (reference range, 90–129 mg/dL [30]) of 101 mg/dL one year after the first infusion. His HDL-cholesterol level markedly decreased, from 72.7 to 51.8 mg/dL at the time of the first infusion after the baseline results, and it gradually increased to 67.3 mg/dL one year after the first infusion. His parents reported that diet, lifestyle, or physical activities were the same as usual before and during the study period and afterward. His alkaline phosphatase (ALP) levels were always in the lower normal range [31]; however, they showed a trend of gradual elevation. Other blood laboratory tests results are presented in Table 1.

### 2.4. Anti-Inflammatory and Anti-Atherosclerotic Responses by CB Cell Therapy

**Figure 2:**
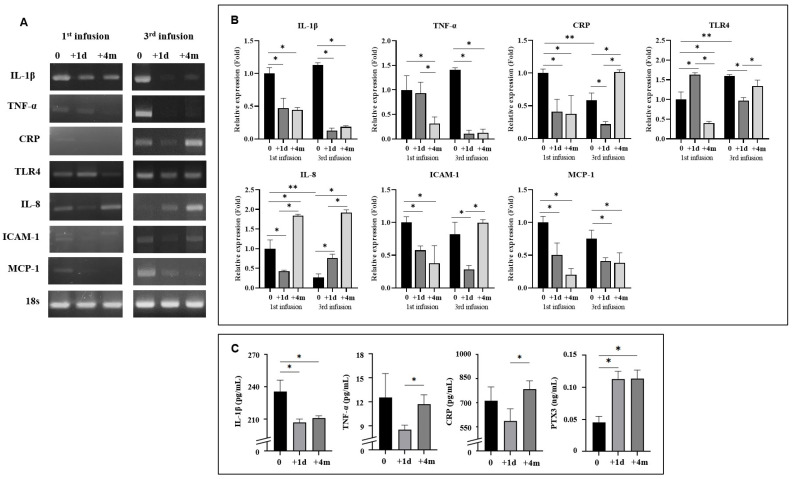
Molecular assays before and after the cord blood cell therapy. (**A**) The mRNA levels of proinflammatory factors, IL-1β, TNF-α, CRP and TLR4; angiogenic factor, IL-8; and atherosclerotic factors, ICAM-1 and MCP-1 are shown. An 18s gene was used as the reference. (**B**) The relative expressions (expressed as their relative folds compared to the baseline value; 0 of first infusion) of mRNA levels for IL-1β, TNF-α, CRP, TLR4, IL-8, ICAM-1 and MCP-1, and (**C**) serum ELISA results of proinflammatory factors, IL-1β, TNF-α, CRP, and innate immune marker, PTX3 are shown in bar graphs. Each peripheral blood sample was drawn on first and third cord blood infusion, before its administration (0), one day after it (+1 d), and four months after it (+4 m). Each experiment was repeated at least three times from the three separate vials, and the data are presented as mean ± standard error. * shows significant difference (*p* < 0.05) between periods following each infusion, and ** shows significant difference when comparing the baseline value of first and third infusion analyzed by independent t-test. Abbreviation: IL-1β, interleukin-1β; TNF-α, tumor necrosis factor-α; CRP, C-reactive protein; IL-8, interleukin-8; TLR4, toll-like receptor 4; ICAM-1, intercellular adhesion molecule-1; and MCP-1, monocyte chemoattractant protein-1.

Assessment of the expression of proinflammatory cytokines at the mRNA level in systemic blood revealed remarkable downregulation of IL-1β, tumor necrosis factor-α (TNF-α), C-reactive protein (CRP), and toll like receptor 4 (TLR4) one day after the first and third infusion, identically. After four months, the lowered levels were maintained in IL-1 β and TNF-α after both the first and third infusion. While gene expression level of CRP kept at lowered level four months after the first infusion, it elevated again at the same time after the third infusion. The gene expression level of TLR4 downregulated four months after both first and third CB infusion, while the level increased the next day after the first infusion and decreased after the third infusion. Their protein expression measured by enzyme-linked immunosorbent assay (ELISA) for the third infusion showed similar findings of continuing downregulation of IL-1β and elevated CRP at four months after treatment. However, the protein expression of TNF-α increased at that time when the gene expression was downregulated. While gene expression of CRP was downregulated one day after therapy, the pentraxin3 (PTX3) level measured using ELISA showed elevation on the same day, which was maintained for four months. Interleukin-8 (IL-8) mRNA levels, known to be angiogenic [18,34] and upregulated in a previous CB trial for cerebral palsy patients [24], continuously increased at 4 months after each infusion (Figure 2).

The mRNA levels of ICAM-1 and monocyte chemoattractant protein-1 (MCP-1), which are atherosclerosis-associated cytokine-coding genes, were downregulated one day after both first and third infusion. The lowered expression level of MCP-1 was maintained four months after both the first and third infusion. The gene expression of ICAM-1 was still at lowered levels four months after the first infusion, although it re-elevated four months after the third infusion (Figure 2).

## 3. Discussion

Studies evaluating the therapeutic efficacy of HGPS in patients are challenging due to its rarity and short lifespan. Corresponding studies have compared the outcomes with those of natural history cohorts [10] or with the pre-treatment rate of changes in the relevant clinical parameters [8]. In the present study, we also employed the change rate of several significant parameters during the pre-treatment period to determine the therapeutic effects of CB treatment. We evaluated similar outcome measures but added blood laboratory tests and molecular mechanism studies as a previous trial on lonafarnib, and set the primary endpoint as weight gain after therapy and the secondary endpoint as decreases in arterial PWV along with carotid artery echodensity and skeletal rigidity [8].

According to the clinical outcomes in the present HGPS patient, CB cell therapy seemingly exerted therapeutic effects in diverse perspectives without side effects that might be related to the intervention. First, the patient showed marked height and weight gain. In children with HGPS, height and weight decrease below the normal third percentile at 15 months and 2 months of age, respectively, and the gaps increase thereafter [35]. In the current study, the baseline anthropometric values of the patient were similar to typical HGPS cases with typical morphology. The patient showed approximately 0.5 cm/year growth before CB cell therapy and 4.3 cm/year and 4.1 cm/year growth for the first and second years after the first infusion of CB cells, respectively. In the present case, facilitated growth was accompanied by a gradual elevation in ALP levels. A study on healthy children showed an increase in ALP around their growth spurts with a median ALP level of >500 IU/L [36]. Although the ALP levels were constantly lower than the reference range (127.2–517.2 IU/L [31]) in this study, the gradual elevation might explain the gain in stature of the patient after CB therapy [37]. Considering a growth hormone trial conducted for HGPS between 2 and 10 years of age, which showed 3.98 cm/year and 3.58 cm/year growth with and without treatment with the hormone, respectively, the growth rate appears to have increased following CB administration. This finding of height gain in the present patient seems meaningful referring other children with HGPS in his age, where two children showed approximately 1–2 cm/year at age 13–14 [38]. Moreover, the patient in the present study gained 1.2 kg and 1.5 kg of body weight in the first and second years following CB therapy, respectively, which contrasts a previous study on HGPS where the gains were 1.01 kg/year and 0.65 kg/year with and without growth hormone therapy, respectively [35]. The rate of weight gain in this patient was also superior to that of children of his age, which was approximately 0.44–0.52 kg/year [39] and 0.5–0.75 kg/year [38].

The musculoskeletal assessment showed extremely low bone density throughout the follow-up, which then decreased after one year, which is consistent with previous reports [35,40]. However, the bone sizes of HGPS children should be considered when adjusting the value taken by dual-energy X-ray absorptiometry (DXA) as HGPS has a lag height of approximately four years [40].

The patient also showed a slight ROM improvement in the proximal joints of his upper extremities. On the other hand, there was a mild decrease in ROM in the knees and right ankle, which might have been caused by a lack of and ambulatory activities. However, this could also be due to rapid growth after therapy. A report on patients with cerebral palsy demonstrated that the highest rate of growth accompanied a significant decrease in joint ROM due to inconsistencies in soft-tissue lengthening and bone growth [41]. Mild cognitive improvement was demonstrated by an IQ score change from below average to average, although patients with HGPS do not tend to have cognitive deterioration [42].

Above all, the most significant finding in the present study was atherosclerosis amelioration by CB cell therapy. As investigated in previous studies, we regarded decreasing PWV benefits in children with HGPS [8,13,43]. Although the decrement was not as large as median PWV reducing to 4.5 m/s from 12.9 m/s, as shown in a previous study [13], and all the values were within the reference range regarding his age (3.9–6.3 m/s, at age 13–14), PWV, which was elevated abruptly during the six months prior to therapy, decreased one year later, indicating vascular stiffness amelioration by CB treatment. The height of the patient, which was markedly shorter than that of his peers, could be an important factor in interpreting PWV values. Immediately before therapy, the PWV was 5.4 m/s, which was higher than the reference range at his height (3.2–5.2 m/s) [26], which had abruptly elevated from 4.6 m/s six months before. Thereafter, it decreased to 5.1 m/s, which was still within the reference range considering the height one year after the first infusion. Although the average IMT still increased after CB treatment, another important marker in atherosclerotic assessment, maximal IMT, which reflects highly advanced stages of atherosclerosis with focal thickening [44], showed a remarkable reduction in its increment rate after therapy, having increased steeply beforehand. These findings might be a significant demonstration of the anti-atherosclerotic effects of CB cells. In a previous study, three and two of 15 patients with HGPS showed abnormal IMT and ABI, respectively [35]. Even though the IMT was not profoundly reduced after therapy, the decreased rate of worsening could be correlated with a decrease in triglyceride levels and an increase in HDL-cholesterol levels. Although one study [45] showed no significant difference between the lipid profiles of HGPS and normal controls, the mean age of the HGPS patients in the study was younger (*n* = 19, mean age 8.0). Hypertriglyceridemia is a widely known risk factor for atherosclerotic cardiovascular diseases owing to its strong association with atherogenic lipoprotein levels [46]. Studies have also shown that a decrease in HDL-cholesterol levels, accompanied by aging, might affect the formation of atherosclerotic plaques in HGPS patients and thus contribute to vascular wall thickening [45]. The patient of present study showed abrupt elevation of serum triglyceride level and depression of HDL cholesterol during one month prior to the first CB infusion without any understandable reason, which seem to have gone along with aggravation of PWV and IMT. The authors regarded the degenerative changes of HGPS was in acceleration when he was enrolled in this study. Thus, reduced triglyceride levels and increased HDL-cholesterol levels after CB therapy could be a meaningful finding in this teenage HGPS patient.

Molecular assays supported anti-inflammatory and anti-atherosclerotic mechanisms as systemic responses to CB cell therapy throughout the trial. These changes were observed uniformly in the mRNA and protein levels of all the biomarkers one day after CB infusion. Only TLR4 gene expression was elevated at that time after the first CB infusion, which was remarkably downregulated four months after. Since we observed increments of TLR4 expression in our previous study of CB therapy for cerebral palsy with a similar study design with immunosuppressant use, this result is regarded to be a concordant finding. The patient did not take sirolimus at the third infusion. Four months after therapy, they showed differential changes in mRNA levels, maintaining low levels of IL-1β, TNF-α, MCP-1 by both the first and third infusion, identically, and maintaining lowered levels of CRP and ICAM-1 by the first infusion and re-elevated levels of these by the third infusion. The expression of proinflammatory markers at the protein level was uniformly downregulated, same as their mRNA level 1 day after CB infusion. Four months after the third infusion, IL-1β remained at a low level, and TNF-α and CRP levels increased again. Therefore, according to the molecular assay results, CB cell administration seems to induce anti-inflammatory and anti-atherosclerotic reactions immediately after therapy. Regarding the different findings between the first and third infusion, administration of sirolimus seemed to have affected the outcomes. Although TLR4 gene expression increased one day after CB infusion when the patient took sirolimus, the levels of TLR4 itself, CRP, and ICAM-1 were downregulated until four months after the first infusion. On the contrary, expression levels of these molecules re-elevated four months after the third infusion when he did not take sirolimus, which can be interpreted as weakening therapeutic effects thereafter. Elevated expression of another cytokine, IL-8, at the mRNA level, was recorded one day after CB administration, which is concordant with our previous clinical studies on children with cerebral palsy that showed elevation of IL-8 protein expression after therapy, and its elevation correlated with neurological recovery [22,24]. In the corresponding animal model research, CB cell therapy induced IL-8-mediated angiogenesis [18]. Elevation in PTX3 expression without CRP expression upregulation one day after CB cell therapy is notable because PTX3 is structurally related to CRP. PTX3, an essential component of humoral innate immunity [47] and an important biomarker in previous clinical trials on CB cell therapy [22,24], may play a role in the mechanism of action of CB cells.

This study may be limited in generalizing the results as it was conducted as a single-case trial. Further clinical research evaluating the therapeutic potency and safety of CB cells is required for HGPS.

## 4. Material and Methods

### 4.1. General Characteristics

A 12-years-and-7-months-old male was referred to the Department of Rehabilitation Medicine at the study hospital. He was diagnosed with HGPS with a *c.1824C>T (p.Gly608Gly)* point mutation in exon 11 of the *LMNA* gene with a typical phenotype. He was born without any specific birth history or abnormal features. At the age of 1 year, growth retardation, alopecia, and lipodystrophy around the abdomen appeared. When he was aged 4 years, he exhibited craniofacial disproportion with micrognathia, prominent eyes, small nose, and scant eyelashes. Echocardiography showed calcification of the aortic and mitral valves and hypertrophy of the internal layer at the internal carotid artery.

On the date he visited the outpatient clinic of the study hospital in October 2018, he had a short stature and typical craniofacial appearance. He also had sclerotic skin that irritated frequently and contractures of multiple joints, including fingers, hips, and knees.

### 4.2. Procedures

The clinical study protocol was approved by the institutional review board (No. 2018-12-031) and uploaded to Clinicaltrials.gov (accessed on 1 October 2021) (NCT03871972). Written consent was obtained from the patient and his guardian.

Before the trial, baseline clinical data were acquired six months prior to therapy. As a therapeutic intervention, infusion of allogeneic CB from unrelated donors was performed three times intravenously at 4-month intervals by the principal investigator. The CB units were supplied by CHA Cord Blood Bank, and their selection criteria were ≥3 matching of HLA -A, -B, and -DRB1 in high resolution and ≥2.0 × 10^7^ total nucleated cells per body weight (kg) (Table S1). Preparation processes, including CB washing, were conducted in the same manner as in our previous clinical studies [48]. To prolong survival of injected allogeneic cells, 2 mg of oral sirolimus (Rapamune^®^, Pfizer, New York, NY, USA) was planned to be administered daily for a week from three days before (D−3) to three days after (D+3) the CB cell infusion. The first infusion was given when the patient was 13 years and 2 months old.

Adverse events were monitored for one year throughout the study and continued thereafter. Clinical variables, including anthropometry, laboratory tests, and joint ROM, were measured on each infusion day and one year after the first infusion. To determine therapeutic efficacy, laboratory test results from one month before and the other clinical data from six months before the first CB infusion were used as baseline reference values. Carotid Doppler measurement findings and ABI were analyzed, which were conducted by a corresponding expert cardiologist six months and immediately before the first CB infusion as well as one year later. DXA and Wechsler Intelligence Scale for Children were also performed immediately before and one year after the first CB administration. Peripheral blood was drawn to measure the levels of IL-1β, TNF-α, CRP, IL-8, TLR4, ICAM-1, and MCP-1. An 18s gene was used as the reference to normalize the level of other genes. We selected blood samples from the first and third CB infusion (immediately before, D+1 day, and D+4 months) [22,24] since each was baseline value and least affected by the irregular administration of oral sirolimus, respectively.

Each blood sample (10 mL collected in sodium heparin vacutainer and peripheral blood mononuclear cells (PBMCs)) was separated from the whole blood by a density gradient centrifugation method using Ficoll-Paque (GE Healthcare, Chicago, IL, USA). The supernatant was obtained without any residue and then measured using an ELISA kit (IL-1β and TNF-α; Biolegend, San Diego, CA, USA, PTX3; R&D system, Mineapolis, MN, USA, CRP; LS Bio, Seattle, WA, USA). The remaining blood was mixed with ethylenediaminetetraacetic acid (EDTA) phosphate buffer saline (PBS), carefully placed on Ficoll-Paque, and centrifuged. After the cloudy PBMC band was collected and washed in EDTA-PBS, the total RNA of PBMCs was extracted using TRIzol^TM^ reagent (Thermo Fisher Scientific, Waltham, MA, USA) according to the manufacturer’s protocol. cDNA was synthesized from total RNA using Maxime RT PreMix (Oligo dT primer) (iNtRON Biotechnology, Seongnam, Korea). Polymerase chain reaction (PCR) was carried out with 100 ng cDNA sample, and the final reaction concentration was as follows: a reaction mixture containing Accupower Taq PCR Master Mix (Bioneer, Daejeon, Korea), 10 pmol of each primer for the appropriate target sequence. The gene-specific primers used are listed in Table S2. After initial denaturation at 9 °C for 1 min, each PCR amplification cycling (annealing and extension) condition was as listed in Table S2, which used a Veriti 96-well thermal cycler (Applied Biosystems, Waltham, MA, USA). PCR products (5–10 μL) were separated by electrophoresis on 1.5% agarose (BD, Franklin Lakes, NJ, USA) in Tris acetate/EDTA buffer containing Redsafe Nucleic Acid Staining Solution (iNtRON Biotechnology, Seongnam, Korea). PCR results were analyzed for the intensity of the PCR band using the ImageJ program.

Each experiment was repeated at least three times using the samples collected and stored in different vials, and statistical comparisons between values from different days were performed using an independent t-test with SPSS version 21.0 software (SPSS, Inc., Chicago, IL, USA). Statistical significance was set at *p* < 0.05.

## 5. Conclusions

We have administered allogenic CB cell infusions to a patient diagnosed with HGPS, and as far as we know, it is the first case to treat a patient with HGPS via allogenic CB cells. Altogether, the study findings suggest that CB cell therapy exerts therapeutic effects on HGPS pathogenesis by ameliorating inflammation and atherosclerosis, which seemed to progress abruptly immediately before CB cell therapy. Clinical parameters showed favorable outcomes, including stature and body weight gain, arterial elasticity, arterial intima-media thickening rate, and lipid profiles. Furthermore, this case study revealed the possibility of cell therapy to control atherosclerotic conditions, a cause of concern in this aging generation. Further studies, if possible, may provide stronger evidence of the effectiveness and feasibility of this optimistic treatment option for patients with HGPS.

## Figures and Tables

**Table 1:** Blood laboratory findings before and after the cord blood cell therapy.

	Reference Value	−1 M	0 M	4 M	8 M	12 M
Triglyceride (mg/dL)	<90 [30]	71	226	172	125	101
HDL cholesterol (mg/dL)	>45 [30]	72.7	51.8	56.7	61.4	67.3
LDL cholesterol (mg/dL)	<110 [30]	132	101	100	128	102
Total cholesterol (mg/dL)	<170 [30]	192	165	183	195	182
ALP (IU/L)	127.2–517.2 [31]	144	164	178	203	201
Platelet (10^3^/μL)	183–370 [32]	478	393	421	425	418
Prothrombin time (sec)	12.0–13.2 [33]	13.9	13.6	13.6	12.9	13.0

Table shows the changes of lab data at 1 month before the therapy, right before the therapy, 4 months, 8 months, and 12 months after the first infusion. Abbreviations: HDL, high-density lipoprotein; LDL, low-density lipoprotein; ALP, alkaline phosphatase.

## Data Availability

The data presented in this study are available upon request from the corresponding author.

## Supplementary Materials

The following are available online at https://www.mdpi.com/article/10.3390/ijms222212316/s1.
