# Supplementary material for: Efficacy of Cord Blood Cell Therapy for Hutchinson–Gilford Progeria Syndrome—A Case Report
Source: Int J Mol Sci. 2021 Nov 15;22(22):12316. doi: 10.3390/ijms222212316 (PMC8619635; doi:10.3390/ijms222212316)
Supplement: Supplementary file 1 [file ijms-22-12316-s001.zip › Figure S2.pdf]

Figure S2. Ankle-brachial index before and after the cord blood cell therapy

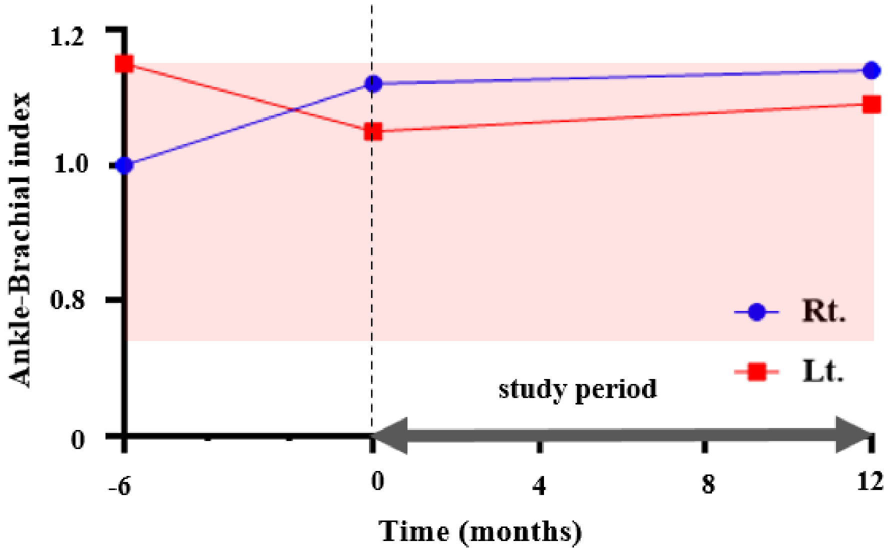

Graph shows the changes of ankle-brachial index (ABI) at 6 months before initiating the therapy, right before the therapy (0 month; initiation of the trial; dotted vertical line) and 12 months after the first cord blood infusion at 0 month. The blue and red dots represent left and right ABIs, respectively. Pink shaded area shows the reference range (0.77-1.15 m/s at age 12-14). Thick bold line with arrows at both ends represents the 12 months of study period starting from the first infusion.
