# Supplementary material for: Efficacy of Cord Blood Cell Therapy for Hutchinson–Gilford Progeria Syndrome—A Case Report
Source: Int J Mol Sci. 2021 Nov 15;22(22):12316. doi: 10.3390/ijms222212316 (PMC8619635; doi:10.3390/ijms222212316)
Supplement: Supplementary file 1 [file ijms-22-12316-s001.zip › Table S1.pdf]

Table S1. General information of infused cord blood units

	1st procedure	2nd procedure	3rd procedure
Date of procedure	May 2019	September 2019	January 2020
No. of infused CB	1 unit	1 unit	1 unit
Total infused volume	27 mL	21.5 mL	22 mL
Pre-thawing TNC (/kg)	4.27×10^7	3.05×10^7	3.59×10^7
Post-thawing TNC (/kg)	3.61×10^7	2.52×10^7	3.30×10^7
Cell viability (CD34+ cell)	96%	88%	82%
% of CD34+ cell	0.49	0.15	0.05
Absolute number of CD34+ cells/μl	101	28	12
Blood type	B+	B+	B+
Immune compatibility*			
Low resolution level	Full match	Full match	Full match
High resolution level	3 mismatch	3 mismatch	3 mismatch

*Immune compatibility was checked by human leukocyte antigen -A, B, and DRB1, which was fully matched at low resolution level and half matched at high resolution level at each of three infusions.

Abbreviation: CB, cord blood; TNC, total nucleated cell.
