# Supplementary material for: Efficacy of Cord Blood Cell Therapy for Hutchinson–Gilford Progeria Syndrome—A Case Report
Source: Int J Mol Sci. 2021 Nov 15;22(22):12316. doi: 10.3390/ijms222212316 (PMC8619635; doi:10.3390/ijms222212316)
Supplement: Supplementary file 1 [file ijms-22-12316-s001.zip › Table S2.pdf]

Table S2. **Gene specific primers and amplification cycling conditions for PCR**

Gene	Prime sequences(5'-3')	Product length (bp)	Amplification cycling conditions (denaturation/annealing/extension)	Total cycles
IL-1β	Sense: AAACAGATGAAGTGCTCCTTCCAGG<br>Antisense: TGGAGAACACCAACTTGTTGCTCCA	391	95℃, 30 sec / 62℃, 30 sec / 72℃, 30 sec	35
TNF-α	Sense: GAGTGACAACCCTGTAGCCCATGTTGTAGCA<br>Antisense: GCAATGATCCCAAAGTAGACCTGCCCAGACT	444	95℃, 30 sec / 68℃, 30 sec / 72℃, 30 sec	35
CRP	Sense: TCCTCGACCCGTGGGTACAG<br>Antisense: ACCAGGGACTGGCTTCCTTC	329	95℃, 30 sec / 62℃, 30 sec / 72℃, 30 sec	35
IL-8	Sense: ATGACTTCCAAGCTGGCCGTGGCT<br>Antisense: TCTCAGCCCTCTTCAAAAACTTCTC	292	95℃, 30 sec / 66℃, 30 sec / 72℃, 30 sec	35
TLR-4	Sense: CGGAGGCCATTATGCTATGT<br>Antisense: TCCCTTCCTCCTTTTCCCTA	141	95℃, 30 sec / 56℃, 30 sec / 72℃, 30 sec	35
ICAM1	Sense: CCCCACAACTTGTCAGCCCC<br>Antisense: GCTCTAGGGTGGGCCTCACA	361	95℃, 30 sec / 62℃, 30 sec / 72℃, 30 sec	35
MCP1	Sense: AGCATGAAAGTCTCTGCCGCC<br>Antisense: GTCCAGGTGGTCCATGGAATC	276	95℃, 30 sec / 68℃, 30 sec / 72℃, 30 sec	35
18s	Sense: GTAACCCGTTGAACCCCATT<br>Antisense: CCATCCAATCGGTAGTAGCG	151	95℃, 30 sec / 58℃, 30 sec / 72℃, 30 sec	35
