# Supplementary material for: Efficacy of Cord Blood Cell Therapy for Hutchinson–Gilford Progeria Syndrome—A Case Report
Source: Int J Mol Sci. 2021 Nov 15;22(22):12316. doi: 10.3390/ijms222212316 (PMC8619635; doi:10.3390/ijms222212316)
Supplement: Supplementary file 1 [file ijms-22-12316-s001.zip › Table S3.pdf]

Table S3. Range of motion in joints before and after the cord blood cell therapy

Joint	Motion Range	Before first infusion		1 year after first infusion	
		Right	Left	Right	Left
Shoulder	Flexion-Extension	240°	**230**°	240°	**240**°
	Abduction-Adduction	210°	**200**°	210°	**210**°
	External rotation-Internal Rotation	180°	180°	180°	180°
Elbow	Flexion-Extension	**130**°	**130**°	**140**°	**140**°
Wrist	Flexion-Extension	95°	90°	90°	90°
Thumb	Flexion-Extension (MP)	5°	**10**°	0°	**0**°
	Flexion-Extension (PIP)	90°	90°	90°	90°
3rd digit	Flexion-Extension (MP)	100°	110°	100°	110°
	Flexion-Extension (PIP)	90°	90°	90°	90°
	Flexion-Extension (DIP)	70°	70°	70°	70°
Hip	Flexion-Extension	117°	113°	100°	100°
	Abduction-Adduction	60°	60°	60°	60°
	External rotation-Internal Rotation	45°	40°	40°	40°
Knee	Flexion-Extension	**70**°	**75**°	**60**°	**60**°
Ankle	Dorsiflexion-Plantar flexion	**40**°	30°	**25**°	25°
Toe	Flexion-Extension (MP)	30°	30°	30°	30°

The motion range that changed more than 10° at 1 year after therapy are shown in bold prints. The motion range were evaluated right before and one year after the first infusion of cord blood cells. Abbreviations: MP, Metacarpophalangeal; PIP, Proximal interphalangeal; DIP, Distal interphalangeal
