# Supplementary material for: Efficacy of Cord Blood Cell Therapy for Hutchinson–Gilford Progeria Syndrome—A Case Report
Source: Int J Mol Sci. 2021 Nov 15;22(22):12316. doi: 10.3390/ijms222212316 (PMC8619635; doi:10.3390/ijms222212316)
Supplement: Supplementary file 1 [file ijms-22-12316-s001.zip › Figure S1.pdf]

Figure S1. Adverse events after cord blood therapy

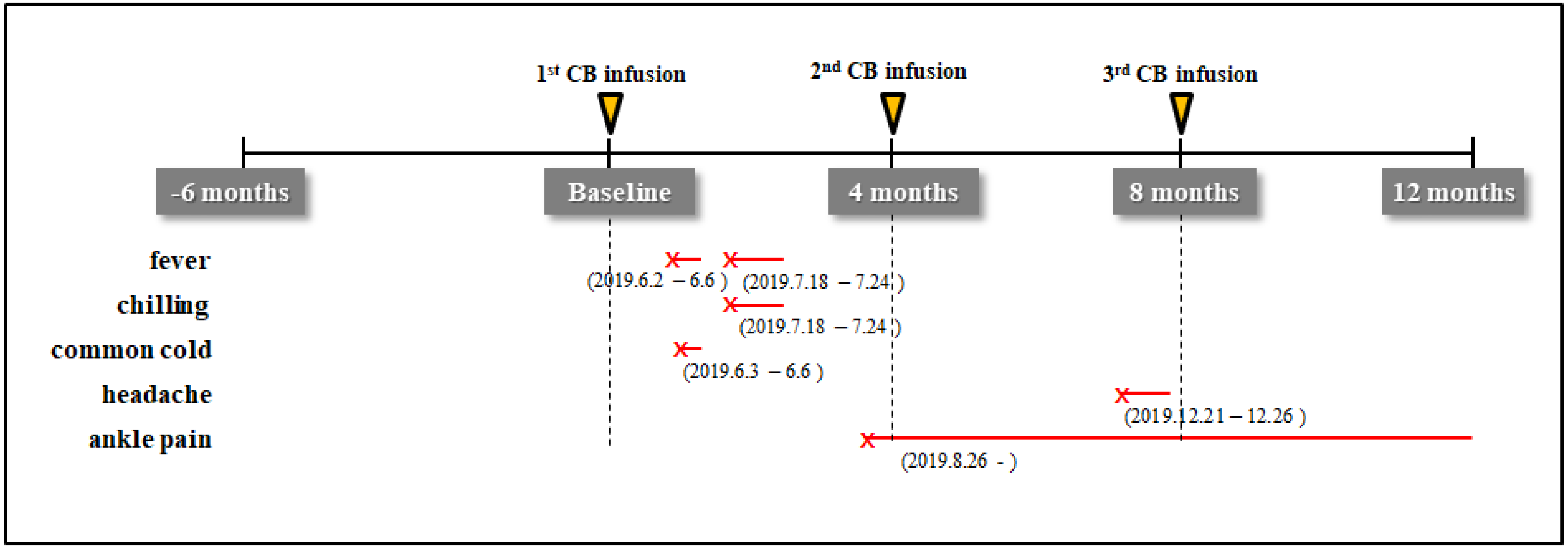

The red X represents the starting date of each adverse event, and the red line represents the duration of each events. Dates in the parenthesis are starting date – end date of the adverse events. The yellow arrows show first, second, and third cord blood infusions, which started from baseline and was given with 4 month-intervals, and was followed until 12 months. The patient showed 6 adverse events during the study period: 2 events of fever, 1 event of common cold, 1 event of chilling senses, 1 event of headache and 1 event of pain in the left malleolus.
